# Prognostic Significance of Early Postoperative Choroidal Detachment in Patients with Congenital Glaucoma Operated with Nonpenetrating Deep Sclerectomy

**DOI:** 10.1155/2024/7127996

**Published:** 2024-07-30

**Authors:** Shaikha H. Aldossari, Konrad Schargel, Ibrahim Aljadaan, Khabir Ahmad, Rakan Gorinees, Nouf Alzendi, Gorka Sesma

**Affiliations:** ^1^ Glaucoma Division King Khaled Eye Specialist Hospital, Riyadh, Saudi Arabia; ^2^ Research Department King Khaled Eye Specialist Hospital, Riyadh, Saudi Arabia; ^3^ Department of Ophthalmology College of Medicine University of Ha'il, Ha'il, Saudi Arabia; ^4^ Pediatric Ophthalmology and Strabismus Division King Khaled Eye Specialist Hospital, Riyadh, Saudi Arabia

## Abstract

**Objective:**

To assess the association between early postoperative choroidal detachment and intraocular pressure (IOP) following nonpenetrating deep sclerectomy in pediatric primary congenital glaucoma.

**Design:**

Retrospective double-arm cohort study. *Setting*. Single center in Saudi Arabia. *Patients*. Seventy-two eyes of 45 patients were evaluated. Primary congenital glaucoma patients aged 0–3 years undergoing nonpenetrating deep sclerectomy as the first procedure from 2014 to 2021 were divided into groups with (*n* = 20) and without (*n* = 52) postoperative choroidal detachment. *Main Outcome Measures*. The primary outcome was complete surgical success, defined as an intraocular pressure below 21 mmHg without medication or additional surgery at 24 months. The intraocular pressure was evaluated in the first 72 hours after surgery and at 1, 3, 6, 12, 18, and 24 months. Kaplan–Meier survival analysis over 24 months was used to evaluate this outcome in both cohorts. The secondary outcome was the time to choroidal detachment resolution.

**Results:**

There was no significant difference in surgical success between choroidal detachment and nonchoroidal detachment groups (*P* = 0.12). Preoperative and 2-year postoperative intraocular pressure was similar between groups, with a significant decrease in intraocular pressure from baseline (*P* < 0.001) in both the groups. The median time to choroidal detachment resolution was 27 days, and 90% of choroidal detachment cases were resolved with medical therapy.

**Conclusions:**

Postoperative choroidal detachment does not appear to significantly impact intraocular pressure or surgical success at 24 months following nonpenetrating deep sclerectomy for primary congenital glaucoma. Choroidal detachment typically resolves within one month of treatment. These findings suggest that transient choroidal detachment has a benign course in patients with primary congenital glaucoma undergoing deep sclerectomies.

## 1. Introduction

Primary congenital glaucoma (PCG) is a sight-threatening condition. The high consanguinity rate in Saudi Arabia is one of the highest incidence rates globally, affecting 1 in 2,500−3,000 live births [1]. CYP1B1 mutations cause most cases [2]. PCG results from prenatal maldevelopment of the trabecular meshwork and Schlemm's canal, causing elevated intraocular pressure, optic nerve damage, and potential blindness if left untreated [3].

The PCG is managed surgically. Treatment options include penetrating procedures, such as goniotomy, trabeculotomy, trabeculectomy, combination surgery, and nonpenetrating procedures, including deep sclerectomy (NPDS) [4–6]. Goniotomy and trabeculotomy are conventional surgical procedures for pediatric glaucoma that demonstrate high success rates in achieving optimal intraocular pressure control in western populations [7, 8]. However, Al-Hazmi et al. reported a 48% failure rate after goniotomy and a 59% failure rate after trabeculotomy in Saudi Arabia [9]. These discrepant outcomes may be attributed to variations in phenotypes, severity, and presence of corneal opacities. The prevalence of corneal opacity is a key factor in choosing appropriate surgical treatment. In Saudi Arabia, many patients with PCG have cloudy corneas, making angle surgery more challenging [10]. Trabeculectomy is an effective treatment but is associated with significant side effects and complications, including flat anterior chambers, bleb-related complications, and endophthalmitis, among others [11].

Roy and Mermoud described nonpenetrating deep sclerectomy (NPDS), a noninvasive glaucoma surgery. It involves partially removing Schlemm's canal to create a new aqueous outflow while preserving the trabeculodescemetic window (TDW). Compared to more invasive surgeries, NPDS reduces overfiltration and complications postoperatively [12–14]. Al-Obeidan et al. demonstrated the safety and effectiveness of NPDS in Saudi PCG patients, achieving a success rate of up to 77.6%. This surgical approach is widely favored for PCG in this region [15].

Common complications of glaucoma surgery include bleb fibrosis, scleral flap closure, hypotony, and retinal detachment [16].

Choroidal detachment (CD) is a recognized complication of glaucoma surgery, with occurrence rates ranging from 5% to 44% after trabeculectomy and 13% after NPDS [17–19]. Factors contributing to CD may include low IOP and inflammation, leading to ocular hypotony and choroidal alterations [20–22]. CD can present with various symptoms, including impaired vision and discomfort. Effective diagnosis often relies on B-scan ultrasound and other imaging techniques, particularly when dealing with cases of corneal opacity in PCG [23].

In adults, high IOP before surgery, low IOP on day one after surgery, and being male increase the risk of CD after trabeculectomy or Ahmed glaucoma valve implantation, but do not affect the success of the surgery [24]. Severe CD often resolves spontaneously without affecting the final IOP [25]. This observation mainly pertains to adult studies, particularly after penetrating surgical procedures. Previous studies reported conflicting postoperative IOP outcomes associated with CD after glaucoma surgery. A retrospective analysis by Yadgari et al. found that early serous CD during trabeculectomy was associated with unfavorable outcomes, including a significantly higher mean IOP over 4 years of follow-up [26]. In contrast, Inatani et al. found that patients with CD after trabeculectomy had a lower postoperative IOP than those without CD [27]. Most studies on CD following glaucoma surgery have predominantly focused on trabeculectomy. However, the association between CD occurrence and surgical outcomes related to IOP control after NPDS remains inadequately explored and clarified, particularly in the pediatric PCG population. To address these gaps in knowledge, our objective was two-fold: first, to ascertain whether CD in children with PCG undergoing NPDS resolves spontaneously, necessitating no additional intervention; and second, to assess whether CD has an impact on IOP outcomes in NPDS within this specific population. Our hypothesis posited that there would be no substantial difference in IOP values between PCG patients who experienced immediate CD following NPDS and those who did not over a 24-month follow-up period. These findings enhance our understanding of how CD influences NPDS outcomes in PCG. These insights may have practical implications in the management and treatment of this condition. This study highlights an uncharted research domain with potential clinical significance.

## 2. Methods

### 2.1. Study Design

This retrospective cohort study was conducted at the King Khaled Eye Specialist Hospital in Riyadh, Saudi Arabia. Patients diagnosed with PCG who underwent NPDS surgery between January 2014 and December 2021 were included in the study. The cohorts comprised those who developed postoperative choroidal detachment (CD) and those who did not up to one week after surgery.

The inclusion criteria for the study were as follows: patients who were 3 years old or younger, diagnosed with primary congenital glaucoma based on the International Consensus Classification of Childhood Glaucoma [28], underwent NPDS as their primary glaucoma surgery, and developed choroidal detachment within one week postoperatively. Standard follow-up visits were scheduled at 24 and 72 hours and at 1, 3, 6, 12, 18, and 24 months. An initial IOP measurement was routinely performed 24 hours after surgery without sedation to detect any significant changes or complications early. We included patients regardless of their postoperative choroidal detachment status if they met the other criteria. Following surgery, patients were divided into groups based on the presence or absence of postoperative CD for analysis.

To address the concern of potentially missing cases of CD that may have resolved spontaneously before the 1-month visit, the postoperative day 1 examination was included to ensure the early detection of any complications. In addition, parents were instructed to report any signs of ocular discomfort or changes in the child's eye condition immediately, allowing for prompt intervention and additional examinations, as needed. Unscheduled visits were conducted if clinically indicated. This follow-up strategy ensured comprehensive monitoring while balancing the practical challenges of frequent sedation in young children.

B-scan ultrasonography was conducted during these visits, and in cases where sedation was not feasible, B-scan ultrasonography was performed with the child restrained on the mother's lap to ensure comprehensive early postoperative monitoring. B-scan ultrasonography was repeated at each visit until the CD resolution was achieved. A specialized department at KKESH administered sedation consisting of pediatricians, anesthesiologists, and nurses adhering to strict protocols for monitored anesthesia care.

Patients were excluded if they did not have a diagnosis of primary congenital glaucoma, had previous glaucoma surgeries, did not undergo B-scan ultrasonography after NPDS, or had incomplete data or follow-up.

### 2.2. Study Outcomes

The primary outcome was complete surgical success of NPDS, defined as an IOP less than 21 mmHg without adjunctive medications or additional surgical intervention for 24 months postoperatively. The secondary outcome was the time to resolution of postoperative CD. We defined resolution as no detectable choroidal detachment on B-scan ultrasonography at the follow-up. We also recorded the number of days from the initial CD diagnosis to the first documented resolution. Postoperatively, the patients were examined until resolution was noted and followed until the end of the study period. If detachment persisted for six months, the time to resolution was censored at 150 days.

### 2.3. Sample Size

We calculated the target sample using Epi Info 7.2 software (CDC, Atlanta, GA, USA). The anticipated occurrence of CD in the unexposed group, which accounts for 50% of cases, and the exposed group, accounting for 100% of cases, is rooted in previous studies that have reported diverse rates of CD after NPDS [21, 26]. With 80% power and an alpha of 0.05, a sample size of 11 eyes in the exposed group and 27 eyes in the unexposed group was required to detect a statistically significant difference between the groups. In this study, 72 eyes of 45 patients were evaluated, including both unilateral and bilateral cases of primary congenital glaucoma. The inclusion of patients with bilateral glaucoma was necessary to ensure a robust sample size and to enhance the generalizability of the findings. Given the rarity of primary congenital glaucoma, obtaining a sufficient number of patients for the study is challenging. Therefore, statistical methods were employed to account for within-patient correlations and to minimize bias due to the inclusion of multiple eyes from the same patient.

### 2.4. Data Collection

Data were extracted from the electronic medical records system to identify patients diagnosed with PCG who met the inclusion criteria. The following patient demographics were collected: sex, operated eye, family history of PCG, age at initial diagnosis, and whether the disease was unilateral or bilateral. Preoperative data collected included information on the presence of corneal opacity and horizontal corneal diameter (HCD), measured with a caliper and central corneal thickness (CCT) using ultrasound pachymetry. The corneal thickness was measured with a probe placed gently on the cornea after administering topical anesthetic drops for precise measurements. Axial length (AL) was measured using A-scan ultrasonography. For this procedure, the ultrasound probe was placed on the cornea with the patient under sedation or general anesthesia, ensuring accurate measurement. The spherical equivalent refractive error was assessed, when possible, and any intraoperative complications were documented. Due to high corneal opacity prevalence, conventional refraction methods were often impossible. We then utilized retinoscopy under general anesthesia to measure preoperative spherical equivalent refraction. Retinoscopy uses light reflexes less affected by media opacities, making it a reliable method for obtaining refractive measurements despite corneal opacities.

Intraoperative data included the date of the operation, the operated eye, the surgeon's name, the dimensions of the scleral flap, the dosage and duration of mitomycin C, and any adverse events that occurred during the procedure. The primary objective of our research was to mitigate the potential impact of surgeon-related bias in NPDS procedures. To accomplish this, we implemented standardized surgical procedures for all operations, ensured that all surgeons were adept at performing pediatric glaucoma surgeries, and statistically accounted for confounding factors. These measures were taken to ensure that our findings accurately reflected the efficacy of NPDS rather than the individual skill of the surgeon. Postoperative data included information on IOP, follow-up date, presence of CD, location and severity of CD in each quadrant, and the frequency of cycloplegic drops used to manage the condition. We evaluated the anterior chamber depth, determined the quadrants involved in CD, and assessed the severity of CD.

### 2.5. Clinical and Paraclinical Assessment

In our investigation, we utilized the Tono-Pen (Reichert Inc., Depew, NY, USA) to measure intraocular pressure (IOP) in the supine position. To ensure patient comfort, topical anesthesia was administered systematically prior to each IOP assessment. During the preoperative phase, IOP measurements were obtained under general anesthesia, both before and after intubation, to monitor anesthesia-related IOP changes. During follow-up visits, which were typically conducted under sedation, IOP was measured using Tono-Pen, and topical anesthesia was applied to facilitate a comfortable and consistent assessment process.

We opted for a B-scan to assess CD instead of relying on fundus image visualization, as most patients exhibited corneal opacities. Skilled ophthalmic technicians used a B-type ultrasonography device, Cinescan S (Quantel Medical SA, Le Breze, France), for CD detection. The ultrasound study was performed while the patient was supine with the probe in direct contact with their closed eyelids after applying a gel coupling agent. All quadrants of the ocular fundus were evaluated. If ultrasound detected hyporeflective suprachoroidal fluid images in conjunction with retinal elevation or choroidal dome-shaped bulk and choroidal thickening, it was classified as CD. The quadrants and locations of the affected ocular fundus were identified. The severity of the CD was assessed in accordance with established criteria from the literature, which categorizes it as mild, moderate, or severe based on the degree of choroidal separation and its effect on ocular structures [21, 26]. Mild CD entails a separation of less than 2 mm, while moderate CD involves a separation of more than 2 mm without contact. Severe CD is characterized by a separation of the choroid measuring over 2 mm. The detached choroid is observed to be pushed towards the posterior pole of the eye, specifically towards the optic nerve. Additionally, the detached choroid extends towards and contacts the choroid in another quadrant within the vitreous cavity, resulting in the formation of kissing choroids. This phenomenon involves choroidal detachment in multiple quadrants, not specifically related to the optic nerve, without causing retinal detachment.

Employing a grading system, we classified preoperative corneal transparency based on the anterior segment structures. This classification system is rooted in the methodology described by El Sayed et al., which categorizes corneal transparency into three categories: clear, moderate, and severe opacity. Corneas deemed clear enable unobstructed observation of the anterior segment structures. Moderate corneal opacity slightly impairs visibility, but the structures remain discernible. Severe opacity, however, precludes observation of the anterior structures without additional illumination [10].

We classified the depth of the anterior chamber by direct observation using a handheld slit lamp under direct and diffuse illumination. A dark black color characterizes a deep anterior chamber, whereas a shallow chamber has a lighter grey shade. This method was chosen for its simplicity and practicality in a clinical setting, especially when examining pediatric patients under sedation. Although the Van Herick method is commonly used to assess anterior chamber depth, it requires precise angle estimation, which can be challenging in sedated young children. Our chosen method allowed for a more straightforward and rapid assessment without compromising accuracy. The cup-disc ratio and refraction were also evaluated in cases in which they were available.

### 2.6. Surgical Procedure

We conducted a comprehensive ophthalmological examination under general anesthesia to evaluate clinical ophthalmological features. We utilized the same surgical approach as described by Khan et al. [29]. Corneal traction sutures were implemented to effectively expose the superior quadrant and ensure adequate exposure, as well as to provide stabilization for the globe during surgical procedures.

The superior nasal bulbar conjunctiva was carefully incised to 6 mm starting at the superior limbus and curving forward. The goal was to avoid buttonholing of the conjunctiva and to minimize trauma. The conjunctiva was prudently detached from the Tenon's capsule and lifted into a single layer. The excess Tenon's capsule was removed. The conjunctival flap was then retracted to expose the sclera. The bleeding was cauterized to keep the surgical field bloodless. Three cellulose sponges soaked in a solution containing 0.4 mg/ml of mitomycin were applied to the exposed sclera for two minutes. Subsequently, the area was irrigated with a balanced salt solution. Using a crescent knife (Eagle Lab Inc., Rancho Cucamonga, California, USA), a superficial scleral flap measuring 5 × 4 mm (40–50% thickness) and a deep flap measuring 4 × 3 mm (90% deep) were created. Meticulous and precise dissection of the deep scleral flap was performed, reaching 1.5 mm from the limbus to the cornea without perforating it. This process continued until we observed spontaneous aqueous outflow, at which point we cut the deep flap. The scleral flap was carefully secured using 10−0 nylon, and 8−0 synthetic absorbable sutures were used to close the conjunctiva. All patients were prescribed topical antibiotics, which included moxifloxacin ophthalmic solution 0.5%, one drop in the affected eye three times daily for seven days, and 1% prednisolone acetate ophthalmic suspension administered in a tapering dosage regimen for four weeks. In the context of managing CD, our approach comprised the administration of topical atropine sulfate 1% ophthalmic solution on a twice-daily basis to attain cycloplegia and minimize ciliary muscle spasms. Furthermore, topical corticosteroids such as prednisolone acetate 1% ophthalmic suspension were prescribed to decrease inflammation and encourage the reabsorption of suprachoroidal fluid. When CD did not resolve promptly with these measures, systemic corticosteroids were taken into consideration to further reduce inflammation. This comprehensive medical management protocol aimed to expedite the resolution of CD and prevent potential complications.

### 2.7. Statistical Analysis

Data were inputted and processed using Microsoft Excel (Microsoft Corp., Redmond, WA, USA) and analyzed using Stata 17 software (Stata Corp. LLC, College Station, TX, USA). Patient demographics and clinical characteristics were summarized using descriptive statistics. Continuous variables were presented as medians and interquartile ranges (IQRs) instead of means and standard deviations (SDs) due to the non-normal distribution of the data. Categorical variables were expressed as frequencies and percentages. Chi-square or Mann–Whitney *U* tests were used to assess group differences. To control for confounding variables and isolate the effects of the factors of interest, we used multivariate and univariate logistic regression models, which presented the results as odds ratios. Kaplan–Meier survival analysis was used to calculate the CD resolution time and surgical success rate curves, which were then compared using the log-rank test. A Cox regression model was used to investigate the association between CD and IOP, with a failure threshold above 21 mmHg. Statistical significance was established at a *P* value of <0.05.

### 2.8. Ethical Considerations

The study protocol (RP 22110-R) was approved by the Research Department of the King Khaled Eye Specialist Hospital and its Ethics Committee, which waived the requirement for informed consent owing to the retrospective study design involving a review of existing medical records. The Declaration of Helsinki strictly adhered to all aspects.

## 3. Results

This retrospective cohort included 72 eyes (52 without CD and 20 with CD after NPDS) of 45 patients. 27 patients had bilateral PCG, and four patients developed bilateral CD.

In the cohort of 45 patients, which included 20 males (44%) and 25 females (56%), 7 male patients (16%) were found to have CD, while 13 males (29%) did not develop it. Among the female patients, 5 (11%) developed CD and 20 (44%) did not.

Table 1 summarizes the demographics, clinical characteristics, and outcomes of the study cohort. Ninety-seven percent of eyes underwent uncomplicated surgery. Ninety-four percent of eyes had moderate to severe corneal opacities. At the initial postoperative assessment, 94% of the eyes had deep anterior chambers compared to 6% of the CD eyes with shallow anterior chambers (*P*=0.01). The CD eyes had higher postoperative topical atropine use (*P*=0.01). Among the CD cases, 12 (60%) involved all four quadrants, with 60% of cases having mild involvement and 40% with moderate involvement. No cases of severe CD, defined as “kissing choroids,” were observed in our cohort.

Intraoperative complications were identified in two instances. One of these involved traction suture slippage resulting in corneal microperforation, which was successfully managed during surgery. Another case involved microperforation of the trabeculodescemetic window, which was promptly addressed during the procedure. To manage this complication, we applied a viscoelastic substance to stabilize the anterior chamber and prevent further leakage. The scleral flap was then carefully sutured with additional 10−0 nylon sutures to ensure a watertight closure. Postoperatively, the patient was closely monitored for signs of hypotony or other complications, and topical antibiotics and corticosteroids were administered to prevent infection and inflammation. This approach successfully resolved the microperforation without further adverse events. Postoperative assessments did not reveal any effect on the incidence of choroidal detachment or the overall success of the surgical outcomes.

Regarding the shape of the pupil in the eyes that developed CD, we observed that pupils generally remained round and reactive. Nevertheless, in instances of severe CD, we occasionally observed mild distortion of the pupil resulting from the anterior shift of the lens-iris diaphragm. This distortion, however, was resolved following the resolution of CD with medical treatment.

At one month, we recorded 360-degree choroidal detachment in 11 eyes (58%).

CD eyes were older at PCG diagnosis than non-CD eyes (*P*=0.047). However, univariate and multivariate logistic regression analyses (Table 2) showed no significant association between age at diagnosis or other clinical variables and CD.

**Figure 1:**
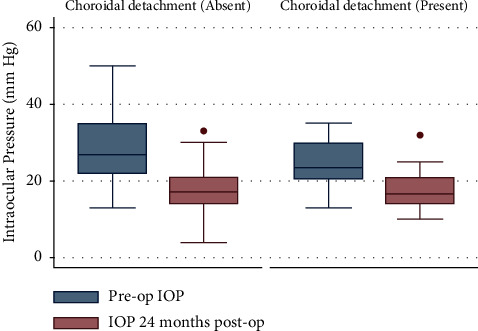
Comparison of preoperative and postoperative intraocular pressure in primary congenital glaucoma patients. The boxplot depicts the IOP over time after surgery. The solid line denotes the median IOP, while the shaded area signifies the interquartile range (IQR). A dot represents a specific data point, indicating an outlier. In both groups, the median IOP at 24 months was less than 21 mmHg. The boxplots contrast the preoperative and 24-month postoperative IOP distributions, revealing substantial reductions in IOP following NPDS, with similar IOP distributions between groups.

IOP was similar between groups preoperatively (*P*=0.07) and 24 months postoperatively (*P*=0.83), with significant decreases from baseline (*P* < 0.001) (Figure 1). There was no significant difference between the two groups (*P*=0.12). At 24 months postoperatively, 75% of the eyes in both cohorts had complete successful surgery, with a median IOP of <21 mmHg. The median IOP reduction in the CD group was 6.5 [0.5; 11.5] mm Hg, whereas the non-CD group displayed a median reduction of 9 [4.5; 18.1] mm Hg.

**Figure 2:**
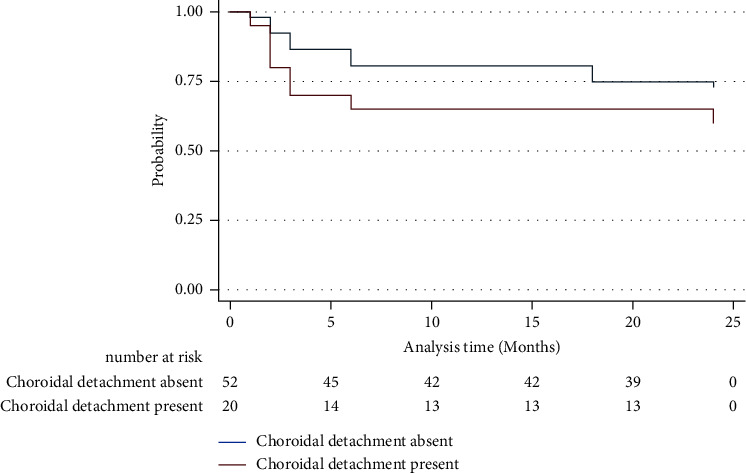
Kaplan–Meier survival curve analysis of intraocular pressure control in patients with primary congenital glaucoma undergoing nonpenetrating deep sclerectomy. The graph illustrates the probability estimates of complete surgical success in patients with PCG who underwent NPDS. Failure was defined as an intraocular pressure (IOP) greater than or equal to 21 mmHg. Patients were divided into two groups based on CD's presence (red line) or absence (blue line) after NPDS.

Kaplan–Meier analysis (Figure 2) indicated a likelihood of complete surgical success, with a 98% chance in non-CD eyes at three months, compared to 80% in CD eyes. At six months, the probabilities were 80% and 53%, and at 24 months, they were 75% and 53%, respectively. Differences in attaining a postoperative IOP of ≥21 mmHg were not significant between the groups (*P*=0.23). Although CD patients had lower probability values for attaining an IOP of >21 mmHg compared with the other groups, the log-rank test revealed no statistically significant differences between the groups (*P*=0.23). The hazard ratio for an IOP above 21 mmHg was 1.3 (95% CI: 0.54–3.16; *P*=0.56), which was not statistically significant.

**Figure 3:**
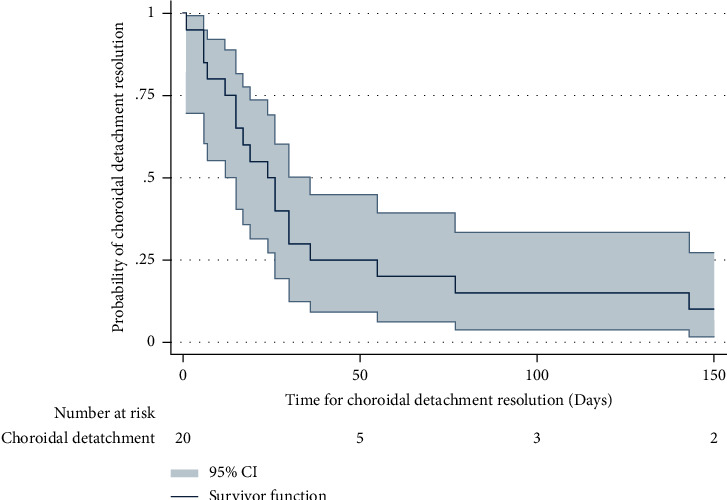
Kaplan–Meier survival estimate for the resolution of choroidal detachment in patients with primary congenital glaucoma who underwent nonpenetrating deep sclerectomy. The Kaplan–Meier survival estimate for CD resolution was 50% at 25 days and 80% at 60 days. After 150 days, two patients had unresolved CD.

The median CD resolution was 27 days. By 25 days and 50 days postoperatively, there was a 50% and 80% chance of resolution, respectively. Two patients showed no recovery across any of the four quadrants after 150 days (Figure 3).

**Figure 4:**
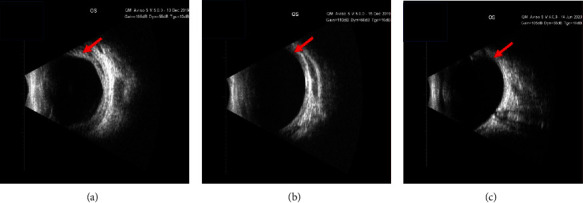
B-scan photographs of choroidal detachment after nonpenetrating deep sclerectomy in a patient with primary congenital glaucoma. (a) B-scan of the eye two days after surgery showing 360-degree CD involving the posterior pole. The red arrow indicates choroidal thickening and hyporeflective images in the outer choroidal layers, consistent with CD. (b) B-scan of the eye seven days after surgery showing decreased CD compared with two days postoperatively. (c) B-scan of the eye 37 days postoperatively showing resolved CD.

The B-scan images shown in Figure 4 illustrate the detection of CD and its subsequent resolution following a four-week course of medical treatment, which includes the following:

Topical atropine sulfate 1% ophthalmic solution: administered twice daily to achieve cycloplegia, diminish ciliary muscle spasm, and deepen the anterior chamber.

Topical prednisolone acetate 1% ophthalmic suspension: administered four times daily to alleviate inflammation and facilitate the reabsorption of suprachoroidal fluid.

Systemic corticosteroids: in cases where CD did not show prompt resolution with topical treatments, oral prednisolone was administered at a dosage of 1 mg/kg/day, with tapering based on the clinical response to further alleviate inflammation.

## 4. Discussion

Our analyses revealed no significant association between CD and changes in IOP at 24 months after NPDS in pediatric patients with PCG. However, the regression analysis revealed a connection between 24-month IOP and CD occurrence. The presence of CD may have impacted early postoperative IOP measurements, but as it resolved over time with treatment, the IOP differences between the groups lessened, resulting in similar mean IOPs at the 24-month follow-up. The model accounted for factors such as baseline IOP, age during surgery, and corneal opacity, revealing connections between IOP at 24 months and CD. This suggests that the initial CD could have influenced long-term IOP control indirectly, possibly through alterations in ocular physiology or postoperative healing mechanisms. Patients with CD may require more intensive postoperative management, including medication adjustments or additional interventions, which can influence long-term IOP outcomes. The regression analysis emphasizes the importance of careful postoperative care in these eyes.

The overall complete surgical success rate (75%) was comparable between the two cohorts.

Our results align with those of Sato et al., who similarly observed no significant influence of CD on surgical outcomes following 360-degree trabeculotomy [30]. This implies that CD may not directly affect postoperative IOP in pediatric patients with PCG and exerts a minimal impact on surgical outcomes in this population. In contrast to our results, one study reported higher postoperative IOP with CD, whereas another study found lower IOP with CD following trabeculectomy in an adult population [26, 27]. These contradictory results highlight the need for further research to examine the relationship between CD and IOP across different surgical procedures and patient populations, including pediatric patients with congenital glaucoma.

The risk of a TDW rupture, resulting in rapid globe decompression, is acknowledged in NPDS and may contribute to CD. The rapid decompression can lead to a sudden drop in the IOP, resulting in iris prolapse and the anterior displacement of the lens-iris diaphragm, causing the pupil to move upward. To minimize this risk, precise surgical techniques were utilized to maintain the TDW's integrity. In situations where microperforation or unintentional rupture was suspected, prompt intraoperative measures were taken, such as applying viscoelastic substances and securely suturing the scleral flap, to stabilize the anterior chamber and prevent further complications. Postoperative monitoring included close observation for signs of hypotony or iris prolapse, with prompt intervention as necessary. Despite these precautions, minor undetected ruptures or excessive filtration may have contributed to CD in some cases. This emphasizes the significance of diligent intraoperative and postoperative management to reduce the risk of such complications.

CD can result from various factors, including IOP elevation, inflammation, trauma, and hypotony [27]. The discrepancy between the studies may reflect differences in patient populations, surgical techniques, follow-up intervals, methods for assessing CD, and IOP measurements. While we exclusively examined NPDS in a PCG population with a 24-month follow-up, Lee et al. and Chen et al. focused on adult patients undergoing trabeculectomy with shorter (6–12 month) follow-up periods [31, 32]. Our research provides novel data on CD-related NPDS outcomes in pediatric patients with glaucoma. The choice of glaucoma surgery and the underlying diagnosis may modulate the IOP response to postoperative CD. Our results reveal the need for procedure-specific and population-specific evidence of the IOP effects of CD in patients with glaucoma. More extensive controlled studies are needed to clarify the significance of CD in this population. In our study, choroidal detachment (CD) occurred in all quadrants, without an identifiable restriction pattern to specific quadrants.

Choroidal detachment (CD) was resolved with medical therapy in 18 of the 20 eyes (90%). The median time to resolution was 27 days, with a 50% probability of 25 days and an 80% probability of 50 days postoperatively. Our results are consistent with those of previous studies showing self-limited CD resolution within 3-4 weeks [21, 33].

Topical atropine and corticosteroids may facilitate the treatment of choroidal detachment (CD). Atropine thickens the ciliary muscles and body, deepens the anterior chamber, and rotates the ciliary body posteriorly [34, 35]. Steroids reduce inflammation and increase intraocular pressure, thereby aiding in CD resolution [36, 37].

Although fundus photography optimally tracks choroidal detachment (CD) progression, ultrasound is the preferred modality for follow-up when media opacity is present. Ultrasound biomicroscopy can also be used to evaluate the anterior segment, peripheral retina, and choroid in these patients.

The B-scan images (Figure 4) depicted choroidal thickening, rather than classic CD. Choroidal thickening is a prevalent postoperative occurrence following globe decompression during glaucoma surgery, and it typically resolves as IOP stabilizes.

Our study showed that 80% of eyes with presumed CD had deep anterior chambers, which is atypical for classic CD, where shallow anterior chambers are more common. This discrepancy suggests that what we identified as CD may represent choroidal thickening due to transient hypotony and subsequent recovery of IOP, rather than genuine CD. Additional research utilizing the state-of-the-art imaging procedures can greatly improve our comprehension of choroidal thickening and facilitate the detection of CD by exposing fluid accumulation in the space above the choroid.

Our observation that CD is associated with deep rather than shallow postoperative anterior chambers contrasts with prior evidence [38]. This may reflect the nonpenetrating procedure, severity of CD (shallow to moderate), and natural pediatric tissue elasticity minimally affecting the anterior segment anatomy. However, a detailed examination of the anatomy of the anterior chamber using ultrasound biomicroscopy could provide further insight [39]. Further investigation is warranted beyond the scope of this study.

Although CD is a potential complication of glaucoma surgery, our findings suggest that most cases follow a benign course and spontaneously resolve with medical treatment. These promising outcomes underscore the significance of vigilant monitoring and appropriate management to expedite the timely resolution of CD after NPDS.

### 4.1. Limitations

This study has several limitations, including its retrospective, nonrandomized, single-center design with multiple surgeons, which may introduce bias. A prospective, multicenter, randomized controlled trial would provide high-quality evidence that would be less susceptible to these biases. The relatively small sample size limited the generalizability of our results. The 24-month follow-up may have missed long-term intraocular pressure changes and choroidal detachment effects. Our focus on pediatric patients with PCG undergoing NPDS means that the results may not extend to other populations, glaucoma types, or procedures. In this study, both eyes of the same patient were included in the analysis when eligible. We recognize that this approach might introduce bias due to the possible correlation between the two eyes of a single patient. Future investigations may benefit from randomly selecting a single eye when both are qualified to minimize this potential bias.

One limitation of our study was that we did not assess conjunctival bleb morphology. Which could have provided valuable information on factors influencing the presence and resolution of choroidal detachment. While our focus was on intraocular pressure and CD, future research should incorporate an analysis of conjunctival morphology to gain a comprehensive understanding of its impact on surgical outcomes in PCG.

## 5. Conclusion

We found no significant association between CD and IOP changes at 24 months after NPDS in pediatric patients with PCG. Based on our findings, CD may not have a direct impact on postoperative IOP in pediatric patients with PCG. In addition, our research suggests that CD minimally influences surgical outcomes in this population. CD was resolved with medical therapy in 90% of cases. The median time to resolution of choroidal detachment was 27 days, highlighting the need for close monitoring and appropriate management after surgery. These results can help inform clinical decision-making and improve patient outcomes in this population.

## Figures and Tables

**Table 1:** A comparison of patient demographics, clinical features, and test outcomes based on the existence of choroidal detachment.

		Choroidal detachment		Total (%)	*P* value
		Absent	Present		
		*n* (%)	*n* (%)		
Eyes		52 (72)	20 (28)	72 (100)	

Gender	Male	21 (40)	10 (50)	31 (43)	0.46
	Female	31 (60)	10 (50)	41 (57)	

Eye operated	OD	30 (58)	7 (35)	37 (51)	0.09
	OS	22 (42)	13 (65)	35 (49)	

Family history	Yes	19 (37)	5 (25)	48 (67)	0.36
	No	33 (63)	15 (75)	24 (33)	

Preoperative corneal opacity	Clear	2 (4)	2 (10)	4 (6)	0.06
	Moderate	44 (84)	11 (55)	55 (76)	
	Severe	6 (12)	7 (35)	13 (18)	

Intraoperative complications	Yes	2 (4)		2 (3)	0.38
	No	50 (96)	20 (100)	70 (97)	
Anterior chamber depth at first follow-up	Shallow		4 (20)	4 (6)	0.01
	Deep	52 (100)	16 (80)	68 (94)	

Topical atropine	Yes	3 (6)	15 (75)	18 (25)	0.01
	No	49 (94)	5 (25)	54 (75)	

Quadrants involved at first follow-up	One		4 (20)	4 (20)	
	Two		4 (20)	4 (20)	
	Four		12 (60)	12 (60)	

Severity of choroidal detachment	Shallow		12 (60)	12 (60)	
	Moderate		8 (40)	8 (40)	
	Severe				

		Median [IQR]	Median [IQR]		

Age of PCG diagnosis (months)		0 [0, 2]	1 [0, 5]		0.047
Preoperative HCD (mm)		12.5 [12, 13.5]	12.5 [12.5, 14]		0.23
Preoperative CCT (microns)		745 [680, 870]	774 [625, 862]		0.72
Preoperative IOP (mmHg)		26.5 [22, 34]	23.5 [20.5, 30]		0.07
IOP 24 hours postoperative (mmHg)		15 [10, 18]	10 [6.5, 18]		0.27
Preoperative axial length (mm)		20.8 [20.2, 22.6]	21.5 [20.8, 23.7]		0.10
Preoperative SE (diopters)		−2 [−2.5, −1.5]	−2.75 [−5.5, −0.5]		0.29

HCD: horizontal corneal diameter; CCT: central corneal thickness; IOP: intraocular pressure.

**Table 2:** Logistic regression analysis of factors associated with the presence of choroidal detachment after NPDS in patients with primary congenital glaucoma.

	Univariate		Multivariate	
	OR (95% CI)	*P* value	OR (95% CI)	*P* value
Gender	0.7 (0.24–1.91)	0.46	0.8 (0.03–1.93)	0.92
Age of diagnosis (months)	1.1 (0.95–1.35)	0.16	1.0 (0.54–1.84)	0.99
Preoperative HCD	1.4 (0.85–2.24)	0.20	1.6 (0.30–9.07)	0.57
Preoperative CCT	1.0 (1.00–1.01)	0.37	1.0 (0.97–1.00)	0.10
Preoperative axial length	1.2 (0.89–1.60)	0.25	0.4 (0.09–1.84)	0.24
Preoperative IOP	0.9 (0.86–1.00)	0.06	1.0 (0.80–1.26)	0.98
IOP 24 months after NPDS	1.1 (1.00–1.17)	0.05	1.3 (0.95–1.86)	0.09
Topical atropine	1.1 (0.67–1.82)	0.72	1.1 (0.68–1.85)	0.65
Preoperative corneal opacity	1.0 (0.85–1.07)	0.45	1.0 (0.85–1.06)	0.35

NPDS: nonpenetrating deep sclerectomy; HCD: horizontal corneal diameter; CCT: central corneal thickness; IOP: intraocular pressure.

## Data Availability

The data used to support the findings of this study are available from the corresponding author upon request.

## Abbreviations

PCG: Primary congenital glaucoma
IOP: Intraocular pressure
NPDS: Nonpenetrating deep sclerectomy
TDW: Trabeculodescemetic window
CD: Choroidal detachment
IQR: Interquartile range
HCD: Horizontal corneal diameter
CCT: Central corneal thickness.
